# Accelerated regression-based summary statistics for discrete stochastic systems via approximate simulators

**DOI:** 10.1186/s12859-021-04255-9

**Published:** 2021-06-23

**Authors:** Richard M. Jiang, Fredrik Wrede, Prashant Singh, Andreas Hellander, Linda R. Petzold

**Affiliations:** 1 Department of Computer Science, University of California, Santa Barbara, Santa Barbara, USA; 2 Department of Information Technology, Uppsala University, Uppsala, Sweden

**Keywords:** Approximate Bayesian Computation, Summary statistics, Discrete stochastic reaction systems, Biochemical reaction systems, Gillespie algorithm

## Abstract

**Background:**

Approximate Bayesian Computation (ABC) has become a key tool for calibrating the parameters of discrete stochastic biochemical models. For higher dimensional models and data, its performance is strongly dependent on having a representative set of summary statistics. While regression-based methods have been demonstrated to allow for the automatic construction of effective summary statistics, their reliance on first simulating a large training set creates a significant overhead when applying these methods to discrete stochastic models for which simulation is relatively expensive. In this *τ* work, we present a method to reduce this computational burden by leveraging approximate simulators of these systems, such as ordinary differential equations and *τ*-Leaping approximations.

**Results:**

We have developed an algorithm to accelerate the construction of regression-based summary statistics for Approximate Bayesian Computation by selectively using the faster approximate algorithms for simulations. By posing the problem as one of ratio estimation, we use state-of-the-art methods in machine learning to show that, in many cases, our algorithm can significantly reduce the number of simulations from the full resolution model at a minimal cost to accuracy and little additional tuning from the user. We demonstrate the usefulness and robustness of our method with four different experiments.

**Conclusions:**

We provide a novel algorithm for accelerating the construction of summary statistics for stochastic biochemical systems. Compared to the standard practice of exclusively training from exact simulator samples, our method is able to dramatically reduce the number of required calls to the stochastic simulator at a minimal loss in accuracy. This can immediately be implemented to increase the overall speed of the ABC workflow for estimating parameters in complex systems.

**Supplementary Information:**

The online version contains supplementary material available at 10.1186/s12859-021-04255-9.

## Background

In recent years, stochasticity has been shown to play a crucial role in many molecular biological processes such as genetic toggle switches [1, 2] and robust oscillators [3]. Systems biologists will often model these stochastic biochemical reaction systems using continuous-time, discrete-space Markov Chains [4], which allow one to capture stochasticity in a system caused by the limited availability of certain reactants, such as transcription factors. A critical step in building an accurate mechanistic model of these stochastic systems is calibrating the kinetic rate constants, to experimental data. While efficient methods exist for parameter estimation using maximum likelihood or Bayesian inference for similar models, for these discrete stochastic models, the intractability of the likelihood function forces researchers to rely on the growing class of Likelihood-Free Inference (LFI) methods [5–7], which depend only on the availability of a model simulator. Recently, Approximate Bayesian Computation (ABC) [8, 9] has become one of the most popular LFI methods for discrete stochastic models due to its simplicity and demonstrated effectiveness.

### Parameter estimation for biochemical systems

In this section, we briefly describe parameter estimation in the context of biochemical reaction systems. Given a biochemical reaction system describing *M* reactions among *N* biochemical species, a single reaction can be specified in the form

$$\begin{aligned} A + B \mathop{\to }\limits^{f(\theta )} C. \end{aligned}$$

where $f(\theta )$ is the kinetic rate function, parameterized by $\theta$, quantifying the rate at which the reaction occurs. In the common situation where $f(\theta ) = \theta$, it is commonly referred to as a kinetic rate constant. The dynamics of the reaction system can be described using numerous mathematical methods such as ordinary differential equations (ODEs), stochastic differential equations (SDEs) and Markov processes. Provided with an experimentally observed dataset, $X_o$, measuring the evolution of the *N* species over time, parameter estimation is concerned with calibrating all of the unknown kinetic rate parameters $\theta$ so that the resulting model replicates the observed data.

A large set of methodologies exist for parameter estimation of biochemical systems, especially when the dynamics are modeled using ODEs. Extensive reviews of some of the prominent optimization, metaheuristic, and Bayesian schemes are detailed in [10–13]. In this work, we are interested in Bayesian parameter estimation when the dynamics of the biochemical system exhibit intrinsic stochasticity and are modeled using a continuous time, discrete-space Markov process [4]. The Bayesian approach allows us to quantify uncertainty in our estimates by using Bayes formula to compute the posterior distribution over the kinetic rate parameters,

1 $$\begin{aligned} p(\theta | X_o) = \frac{p(X_o | \theta )p(\theta )}{p(X_o)}. \end{aligned}$$

A major challenge to this is that the intractability of the resulting likelihood, $p(X | \theta )$, limits algorithms to those in the class of Likelihood-Free Inference (LFI) methods [5–7], such as Approximate Bayesian Computation, which we present below.

### Approximate Bayesian computation

Given a prior over parameters $p(\theta )$ and a stochastic simulator $p(X | \theta )$, Approximate Bayesian Computation (ABC) approximates the posterior distribution $p(\theta | X) \propto p(X | \theta ) p(\theta )$ using only forward simulations and without computing the likelihood [8]. The basic Rejection ABC is presented in Algorithm 1.
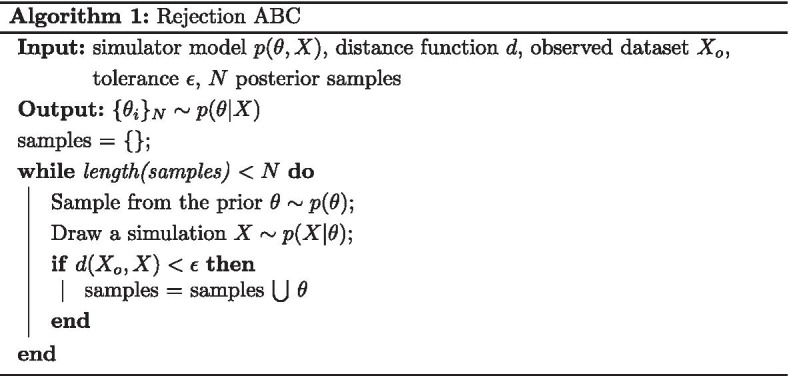


When *X* is high dimensional, comparing exact trajectories often results in very low acceptance rates due to the curse of dimensionality. For this reason, it is standard practice to trade bias for efficiency by first reducing the dimensionality of *X* using a set of *summary statistics*, *S*(*X*), and subsequently comparing trajectories using $d(S(X_o), S(X))$, where *d* is a user selected distance function. This can lead to much higher acceptance rates, however, selection of an appropriate *S*(*X*) for any given model can be difficult.

### Regression-based summary statistics

The performance of ABC is highly dependent on having an effective set of summary statistics for the experimental data, which becomes increasingly difficult for domain experts to hand-select as the dimensionality of the problem grows [8]. For stochastic biochemical reaction systems, where data is often in the form of sample paths of molecular species over time, this is a common issue due to complexity of trajectories where simple means and correlations may not effectively capture the features. For this reason, significant focus has recently been given to the automatic learning of summary statistics from model simulations, which we will refer to as regression-based summary statistics.

Fearnhead and Prangle [14] formulate the problem of regression-based summary statistics for ABC as a least squares estimation of the posterior mean:

2 $$\begin{aligned}&S(X) = {\mathbf {E}}[\theta | X] = f_{\Phi }(X) \end{aligned}$$

3 $$\begin{aligned}&\theta | X \sim {\mathcal {N}}(f_{\Phi }(X), 1) \end{aligned}$$

4 $$\begin{aligned}&\theta = f_{\Phi }(X) + \epsilon , \end{aligned}$$

where $f_{\Phi }$ is an arbitrary expressive function and $\epsilon$ is standard normal noise. The parameters of $f_{\Phi }$ are fit using maximum likelihood on a simulated dataset ${\mathcal {D}} = \{(\theta _0, X_0) \ldots (\theta _N, X_N)\}$ drawn from the model $p(\theta , X)$. While initially proposed as a linear $f_{\Phi }(X)$ for each parameter, nonlinear Neural Network architectures have shown promise in producing accurate results [15]. For discrete stochastic models, Akesson et al. [16] show that Convolutional Neural Networks (CNNs) tend to outperform other architectures. The general procedure for this is detailed in Algorithm 2.
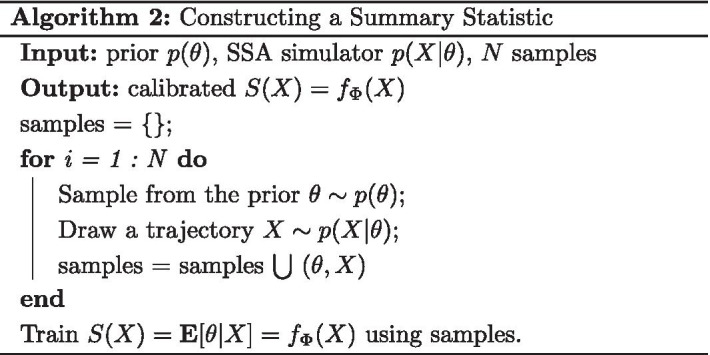


A major bottleneck of regression-based summary statistics is their requirement to first draw a large number of simulations *N* to train accurate summary statistics. For discrete stochastic models which rely on expensive simulators such as Gillespie’s stochastic simulation algorithm (SSA) [17] for generating exact trajectories, this step introduces a significant overhead in ABC. Fortunately, many faster approximate simulators exist for biochemical reaction systems, such as Ordinary Differential Equations (ODEs) in the form of the reaction rate equations (RRE), the Chemical Langevin Equation (CLE) [18], or *τ*-Leaping [19]. However, training a regression-based summary statistic using an approximation will inevitably lead to bias due to the unknown approximation error as the summary statistics will learn incorrect features.

In this work we propose to use data driven machine learning models to train approximate summary statistics for discrete stochastic models using a mix of samples from an approximate simulator and the SSA. This is done with the aim of significantly lowering the computational cost while also mitigating the potential introduced bias in a black-box way. The key insight used for this is that, although the quality of an approximate simulator can vary significantly as we move around parameter space, in many parts it is sufficiently accurate, but also often unknown. To take advantage of this, we train an approximate ratio estimator to inform when the approximation is significantly different and thus when we need to simulate using the SSA to prevent bias. In the following, we demonstrate the ability for our algorithm to effectively reduce the number of expensive SSA calls made, while maintaining accuracy of the learned summary statistics.

### Related work

The use of multifidelity simulators for Approximate Bayesian Computation has been explored, but under the assumption of the existence of a set of summary statistics. Prescott and Baker [20] construct a similar decision process for using multifidelity simulators within ABC-MCMC and ABC-SMC algorithms. In their method, they derive optimal continuation probabilities from a set of assumptions, while we take the more black-box approach of using Deep Neural Networks and approximate ratio estimators.

Approximate Likelihood Ratios have been used to perform likelihood free inference within both an MCMC and an ABC framework [21–24]. These works have mainly focused on estimating the likelihood ratios within a single model at different parameter points, whereas our focus is on estimating the likelihood ratio between approximate and full models.

## Results

### Approximate summary statistics overview

The goal of our algorithm is to reduce the computational cost of constructing a set of regression-based summary statistics for ABC by leveraging the availability of a single approximate simulator. This is accomplished by our algorithm in two major steps. First, a ratio estimator is trained to distinguish between approximate and SSA trajectories using *M* samples from both simulators. Next, to train the summary statistic, $N - M$ additional samples from the approximate simulator are drawn and passed through the ratio estimator. If the ratio estimator falls below a certain threshold, indicating that it is significantly different than the true model, we resample it using the full simulator, preventing unnecessary resamples from the costly SSA. For complete details see “Methods” and Algorithm 3.

### Experiments

To assess the computational savings of our method, we evaluate our method on four discrete stochastic models of varying complexity and compare to the baseline Algorithm 2 which uses no approximate simulations. We report the total number of SSA calls used to train a summary statistic as opposed to wall clock time due to the highly parallelizable nature of the problem. The baseline method utilizes *N* SSA calls but produces the most accurate summary statistic by definition. Accuracy of the resulting summary statistic is evaluated using normalized posterior mean absolute error $E_\%$ [16] on a large hold out test set of SSA trajectories. We briefly explain $E_\%$ in the following section.

For each experiment, we denote *X* for trajectories that are simulated from SSA and ${\tilde{X}}$ for trajectories simulated from the approximation. Each trajectory is also labeled with $Y = \{0,1\}$ where $Y = 1$ indicates that the trajectory came from the SSA simulator and $Y = 0$ indicates that the trajectory came from the approximate simulator. Errors are reported using 30 replications of training and evaluation. We also plot the predictions of the trained approximate ratio classifier on samples drawn from the approximate simulator for each experiment. The output of this is interpreted as the probability, under the trained ratio estimator, that the approximate trajectory ${\tilde{X}}$ at $\theta$ came from the SSA model, $P(Y = 1 | {\tilde{X}}, \theta )$. Values near 0 or 1 inform the decision to resample using the SSA model, as we know that the true class label for ${\tilde{X}}$ is $Y = 0$. Probabilities near 0.5 indicate that the ratio estimator cannot distinguish between SSA and approximate samples and the approximate does not need to be resampled. Complete details for each experiment can be found in the Supplementary Materials.

All experiments were conducted using the StochSS and gillespy2 [25] packages for simulating biochemical reaction systems. Specifically, for our approximate simulators, ODE trajectories were generated using the adaptive LSODA integrator [26] and *τ*-Leaping trajectories were generated using the adaptive *τ*-Leaping algorithm of [27] under the default package parameters of gillespy2.

### Normalized posterior mean absolute error $E_\%$

We evaluate the performance of our experiments using the normalized posterior mean absolute error $E_\%$ [16], which is defined as,

$$\begin{aligned} E_\% = \frac{{\mathbb {E}}_{\theta \in p(\theta )}|\theta - {\hat{\theta }}|}{{\mathbb {E}}_{\theta \in p(\theta )}|\theta - {\bar{\theta }}|}. \end{aligned}$$

In this setup, ${\hat{\theta }}$ is the posterior mean and ${\bar{\theta }}$ is the prior mean. This quantity can be approximated for a uniform prior *U*(*a*, *b*) over a set of *N* test points as

$$\begin{aligned} E_\% \approx \frac{4}{b - a}\frac{1}{N}\sum _{i = 1}^{N} | \theta _i - \hat{\theta}_{i} |, \end{aligned}$$

where $\hat{\theta}_{i}$ is obtained using the regression based summary statistic, which is trained to predict the posterior mean.

$E_\%$ aims to quantify the information gained in the posterior distribution. A value of $E_\% = 1$ indicates no information gained while values of $E_\% < 1$ indicate relative accuracy improvements. The true value of this quantity depends on the informativeness of observations, which is unknown in general for most problems. For this reason, the quality of different summary statistics are compared relative to each other under the assumption that the SSA trained summary statistic is maximally informative and the ground truth.

### Pure-birth process

The Pure-Birth Process, or homogenous Poisson Process, is a trivial example where the likelihood is tractable and the *τ*-Leaping approximation produces exact trajectories for all parameter values. In a biochemical system, the pure-birth process represents the spontaneous generation of a molecular species at a fixed rate, which, while simplistic by itself, is often a fundamental component in more complex models. The model is described by a single parameterized reaction where *S* denotes an arbitrary biochemical species:

$$\begin{aligned} \phi \mathop{\rightarrow}\limits^{k} S, \end{aligned}$$

with initial condition of $S_0 = 0$. We assign a wide uniform prior $k \sim {\mathcal {U}}(0, 10000)$ and observe the process at times $t = \{0 : 100 : 1\}$. Though trivial, this example explores the ability to learn the correct approximate ratio-estimator, which should always predict around 0.5 due to the exactness of the approximation.

**Fig. 1:**
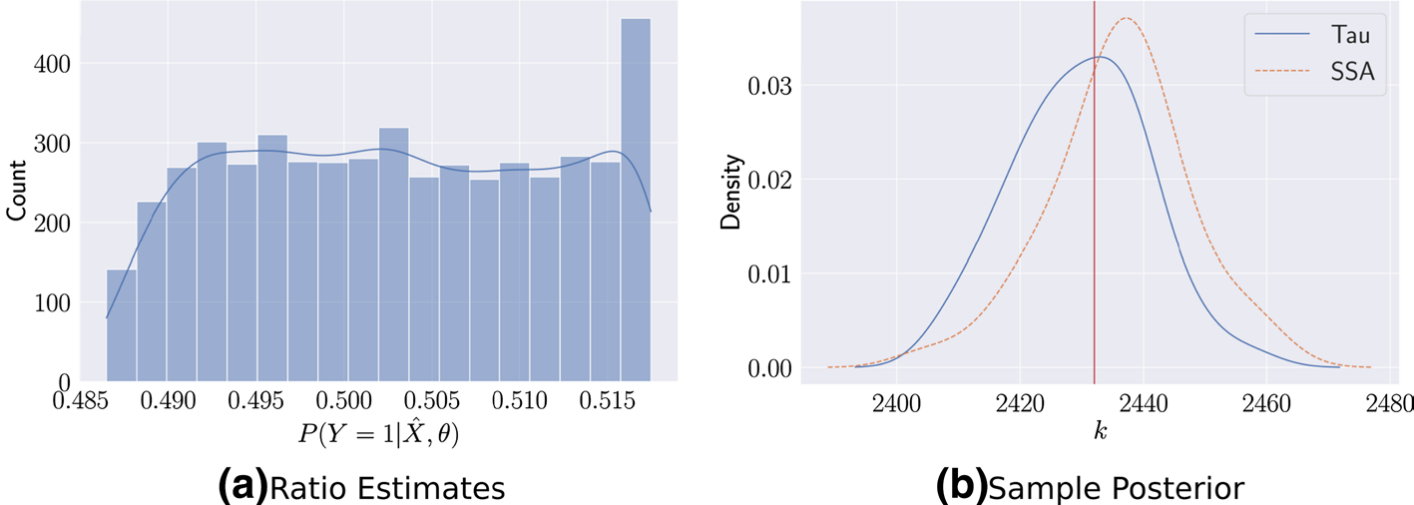
Calibrated Ratio Estimates for the Pure Birth Process **a** The trained ratio estimator captures that the *τ*-Leaping approximation is exact, assigning a probability of  0.5 to all samples. **b** The posterior from both summary statistics captures the ground truth

Figure 1a shows the output of the approximate ratio estimator trained on only $M = 300$ samples from the parameter space and evaluated on 5000 samples from the approximate model. The concentration around 0.5 indicates that the ratio estimator is able to detect that the two models evaluate the same likelihood. Indeed, in Fig. 1b, we see that the posterior distributions using summary statistics trained via only *τ*-Leaping samples or only SSA samples are effectively the same. In this situation, we use a very small amount of samples from the SSA model to build the ratio estimator but otherwise rely entirely on the *τ*-Leaping approximation for no loss in accuracy.

### Lotka-volterra stochastic oscillator

A more challenging and commonly used test problem is the Lotka-Volterra stochastic oscillator. This model describes predator-prey population dynamics and can be modelled as a discrete stochastic system. With $S_1$ representing the count of predators and $S_2$ representing the count of prey, the system is specified via the following set of reactions:

$$\begin{aligned}&S_1 + S_2 \mathop{\rightarrow}\limits^{k_1} 2 S_1 \quad \quad S_1 \mathop{\rightarrow}\limits^{k_2} \phi \\&S_2 \mathop{\rightarrow}\limits^{k_3} 2 S_2 \quad \quad S_1 + S_2 \mathop{\rightarrow}\limits^{k_2} S_2 \end{aligned}$$

with initial populations of $S_1(0) = 50, S_2(0) = 100$. We assign priors,

$$\begin{aligned} & \log (k_{1} ) \sim {\mathcal{U}}( - 6,2)\quad \log (k_{2} ) \sim {\mathcal{U}}(- 6,2) \\ & \log (k_{3} ) \sim {\mathcal{U}}( - 6,2)\quad \log (k_{4} ) \sim {\mathcal{U}}( - 6,2), \\ \end{aligned}$$

and observation frequency following [28] and select a deterministic ODE as our approximating simulator. A key characteristic of this model is that, over the specified prior, only a small region of parameter space leads to consistent oscillations in both the ODE and the SSA models. In most other regions, population explosions are the typical behavior. We train the ratio estimator using $M = 3000$ samples and train the summary statistic with $N = 10^5$ samples. $E_\%$ is evaluated using 300000 hold out SSA test samples.

**Fig. 2:**
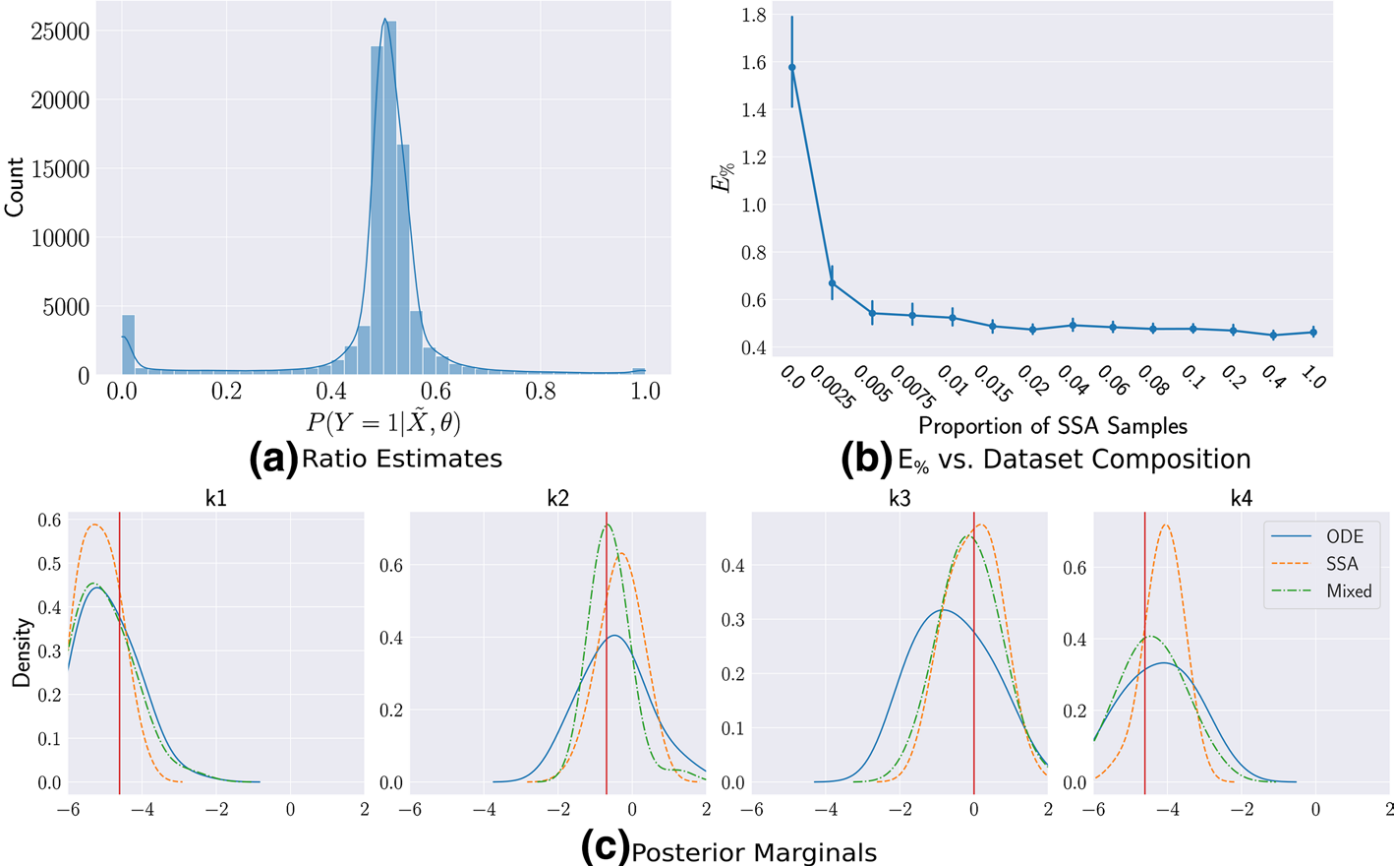
Trained Ratio Estimates for the Lotka-Volterra Stochastic Oscillator **a** The trained $P(Y = 1 | {\tilde{X}}, \theta )$ for the Lotka-Volterra easily classifies many cases, indicated by the peak at the left tail, but remains uncertain for the majority. **b** As the proportion of included SSA calls increase using the ratio estimator, the error quickly falls. Note the nonlinear x-axis, suggesting a very stiff decline in error. **c** Posterior marginals for the four parameters shows that all three summary statistics are able to perform roughly equivalently in the oscillating region

As shown in Fig. 2a, the trained ratio estimator assigns significant mass around 0.5 but with heavy tails, suggesting that some proportion of samples should be resampled using SSA for better accuracy. Figure 2b shows the sensitivity of $E_\%$ as we increase the proportion of SSA samples according to the ratio estimator. In this case, the error rapidly reduces to the level of the full SSA summary statistic by introducing only $1.5\%$ of SSA samples. Assigning an insufficient proportion of SSA samples leads to significantly larger errors.

Figure 2c shows the posterior distribution of the trained summary statistics for a set of observations in the oscillatory regime. All three posteriors are able to capture the true parameters, indicating that for certain parts of parameter space, the ODE and the approximate ratio summary statistic can perform just as well as the SSA trained summary statistic. However, the lower $E_\%$ indicates that globally the mixed summary statistics may perform better. Of note in this example is that, despite the ODE being deterministic, we still obtain good results, demonstrating the robustness of the method to having a perfectly precise ratio estimator.

#### Comparison to random

To evaluate the benefit provided by the ratio estimator, we compare summary statistics trained via our ratio estimator method to summary statistics trained by randomly re-sampling a fixed proportion of approximate samples to SSA samples on the Lotka-Volterra model. Under this setting, each summary statistic is trained using the same proportion of expensive stochastic simulations however how the trajectories are selected differ.

Table 1 shows the $E_\%$ as we increase the proportion of SSA trajectories under these two methods. We see that, while the randomly trained summary statistic is capable of producing comparable results to our ratio estimator approximate summary statistic, it is far less robust, especially when the proportion is small. This can be explained because as we include more random samples, the chance of randomly including the same samples as the ratio estimator becomes much higher. For the Lotka-Volterra model, as it does not require many SSA samples to obtain good performance, as seen from our experiments, this happens relatively quickly.

**Table 1:** Approximate Summary Statistic Median $E_\%$ for Lotka-Volterra for 100 iterations with 90% intervals

% of SSA samples	Random *E*_%_	Ratio estimator *E*_%_
0.25	1.22 [0.44, 2.47]	0.53 [0.42, 1.12]
0.75	0.60 [0.43, 1.89]	0.46 [0.42, 0.85]
1.5	0.46 [0.42, 1.19]	0.45 [0.42, 0.71]
4.0	0.46 [0.42, 0.87]	0.46 [0.42, 0.70]

### Genetic toggle-switch

The Genetic Toggle-Switch is a model for a biological system which exhibits stochastic switching behavior at low-population counts [29]. With *U* and *V* representing two biochemical species which mutually repress the other, the system is described by the following set of reactions:

$$\begin{aligned}&\phi \mathop {\longrightarrow }\limits ^{\frac{\alpha _1}{1 + V^{\beta }}} U \quad \phi \mathop {\longrightarrow }\limits ^{\frac{\alpha _2}{1 + V^{\gamma }}} V \\&U \mathop{\rightarrow}\limits^{\mu } \phi \quad V \mathop{\rightarrow}\limits^{\mu } \phi . \end{aligned}$$

For our study, we set the initial conditions to $U = 10, V = 10$, assign the following priors to the parameters,

$$\begin{aligned}&\alpha _1 \sim {\mathcal {U}}(0, 3) \quad \alpha _2 \sim {\mathcal {U}}(0, 3) \quad \\&\beta \sim {\mathcal {U}}(0, 3) \quad \gamma \sim {\mathcal {U}}(0, 3) \quad \mu \sim {\mathcal {U}}(0, 3), \end{aligned}$$

and use an adaptive *τ*-Leaping solver [27] as our approximate simulator, which in this model produces trajectories with consistently higher population counts than the SSA model. Since these differences correspond to areas that have small population counts, the difference in ensemble results are significant. We train the ratio estimator using $M = 5000$ and train the summary statistic using a budget of $N = 10^5$. $E_\%$ is evaluated using 300000 hold out SSA test samples.

**Fig. 3:**
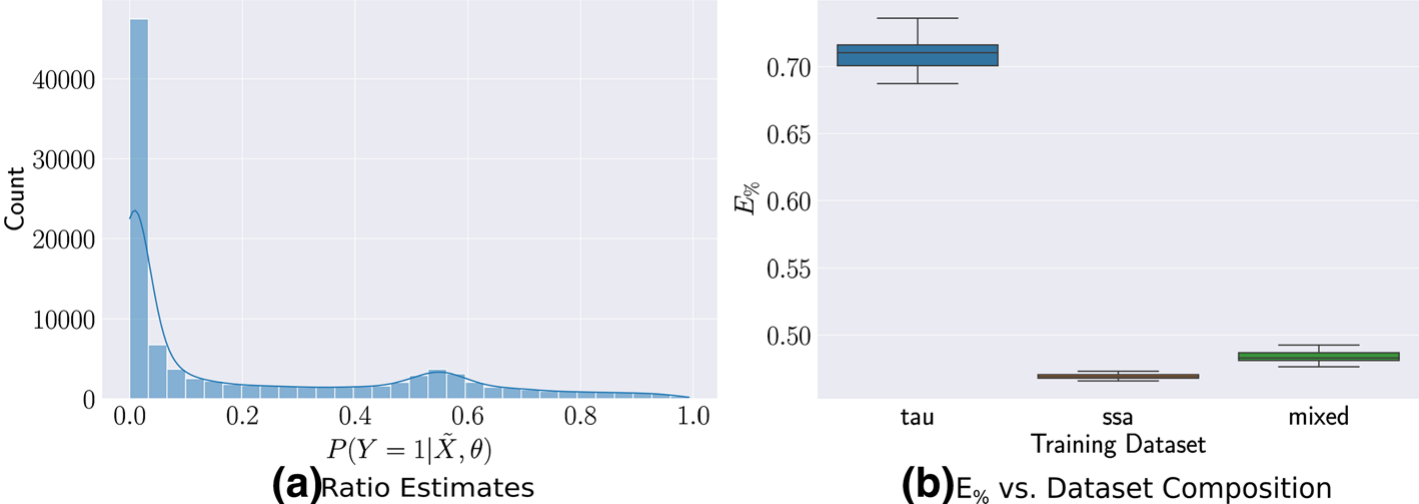
Trained Ratio Estimates for the Genetic Toggle-Switch **a** The trained $P(Y = 1 | {\tilde{X}}, \theta )$ can easily classify most of the cases. **b** The $E_\%$ error only slightly increases by using our mixed training set but still reduces SSA calls significantly

Figure 3a shows the predicted ratios for all $10^5$ low-fidelity samples after training, indicating that the classifier can easily distinguish the correct class of most of the *τ*-Leaping samples. However, as there is still mass near 0.5, using a very small $\rho = 0.01$, we are able to reduce the number of SSA calls by $50\%$ while only losing $2\%$ in $E_{\%}$. Under this prior, though most of the parameter space leads to small population counts, significant portions lead to growth in the populations of *U* and *V*, where the *τ*-Leaping approximation is more accurate. The trained ratio estimator is able to capture this difference and prevent expensive resampling.

### Vilar oscillator

To investigate our method on a larger problem with a questionable approximation, we look at a stable stochastic genetic oscillator [3] modelling a circadian clock. The system is defined with 9 species and 18 reactions controlled by 15 rate constants, and is designed to produce robust oscillations in the presence of intrinsic noise. See the Appendix for further details for the reactions of this model. The Vilar Oscillator is a challenging problem for inference due to oscillations of a certain amplitude being localized to small region of parameter space coupled with the large prior space. We use an ODE model with log-normal noise as our approximation and only observe species *C*, *A*,  and *R* of the system. Under the observational settings for this model, the parameters are generally poorly identified [16]. The ratio estimator is trained using $M = 10000$ and the summary statistic is trained using $N = 200000$. $E_\%$ is evaluated using 300000 hold out SSA test samples.

**Fig. 4:**
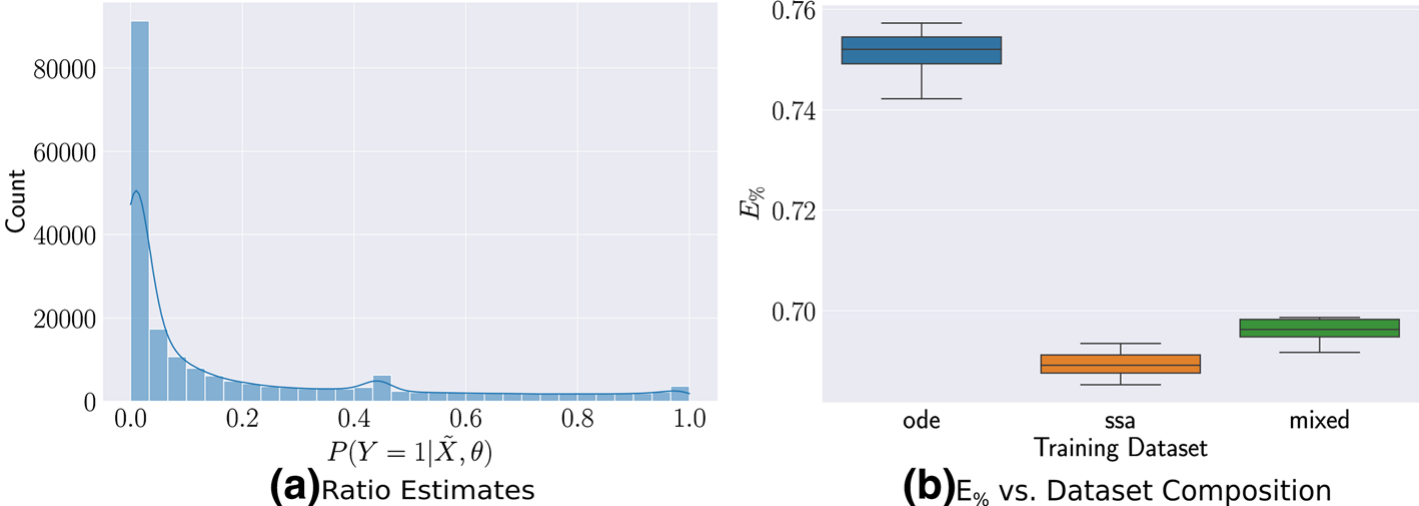
Trained Ratio Estimates for the Vilar Oscillator **a** The trained $P(Y = 1 | {\tilde{X}}, \theta )$ can easily classify most of the cases. **b** The $E_\%$ error only slightly increases by using our mixed training set but still reduces SSA calls significantly

Figure 4a shows that the trained approximate ratio estimator is able to easily classify most of the ODE solutions with added noise, suggesting that the ODE model is a fairly poor approximation. While this model is robust to noise and the mean is captured well by the ODE, at the same time, the log-normal noise does not properly capture the variance and the ratio estimator is able to distinguish the two. This is potentially also useful to diagnose whether an approximation is appropriate to study the model. Nevertheless, due to the addition of noise, the ratio estimator remains uncertain about some areas and, using a $\rho = 0.1$ corresponding to the ratio estimator, we are still able to reduce the number of SSA calls by  $45\%$ and obtain a similar $E_\%$ to that of the full SSA dataset as seen in Fig. 4b. This shows that, even when the approximation is poor, computational savings can still be accomplished while maintaining accuracy by intelligently selecting resamples according to the ratio estimator.

## Discussion

Tables 2 and 3 summarize the primary results for all of the experiments. The results report the average $E_\%$ over 30 replications. Notably, in each case, using our method we are able to train a summary statistic using significantly fewer expensive SSA calls with only a small loss in accuracy. Overall the trained ratio estimator is able to detect when the approximate simulator is good and thus when to lean heavily into the approximate simulator for training.

**Table 2:** Ratio-estimated approximate summary statistic SSA calls

	Total simulations	Approximate total SSA calls	% Approximate reduction in SSA calls
Pure-Birth	30000	0	− 100%
Lotka-Volterra	100000	10000	− 90%
Genetic Toggle-Switch	100000	50000	− 50%
Vilar Oscillator	200000	110000	− 45%

**Table 3:** Approximate summary statistic average $E_\%$

	Approximate only	SSA	Mixed	% Change in *E*_%_
Lotka-Volterra	1.45	0.46	0.48	− 4.3%
Genetic Toggle-Switch	0.71	0.47	0.49	− 2.1%
Vilar Oscillator	0.74	0.68	0.69	− 1.5%

### Practical implementations

While a precise ratio estimator will inevitably lead to an accurate algorithm, we find that in many cases, the ratio estimator for training a summary statistic does not need to be incredibly accurate. In fact, a very expressive ratio estimator may overfit to noise and lead to perfect classification while less expressive ratio estimators can produce a similar level of accuracy in the summary statistic. This is most apparent when we use an ODE as an approximation, where the ratio estimator can quickly learn to discriminate based on the smoothness of solutions. Nevertheless, this can still be useful for summary statistics, as approximate models can often still represent the high-level features. In our examples, we use a variety of Neural Network architectures to learn the ratio estimator, but we find that often, a simple DNN suffices to obtain similar results. For the Lotka-Volterra ODE model, we use a DNN to prevent overfitting to noise, as mentioned above. We use a CNN architecture similar to [16] for the other models where the approximation is stochastic, and suggest a similar approach based on the approximate simulator used.

**Fig. 5:**
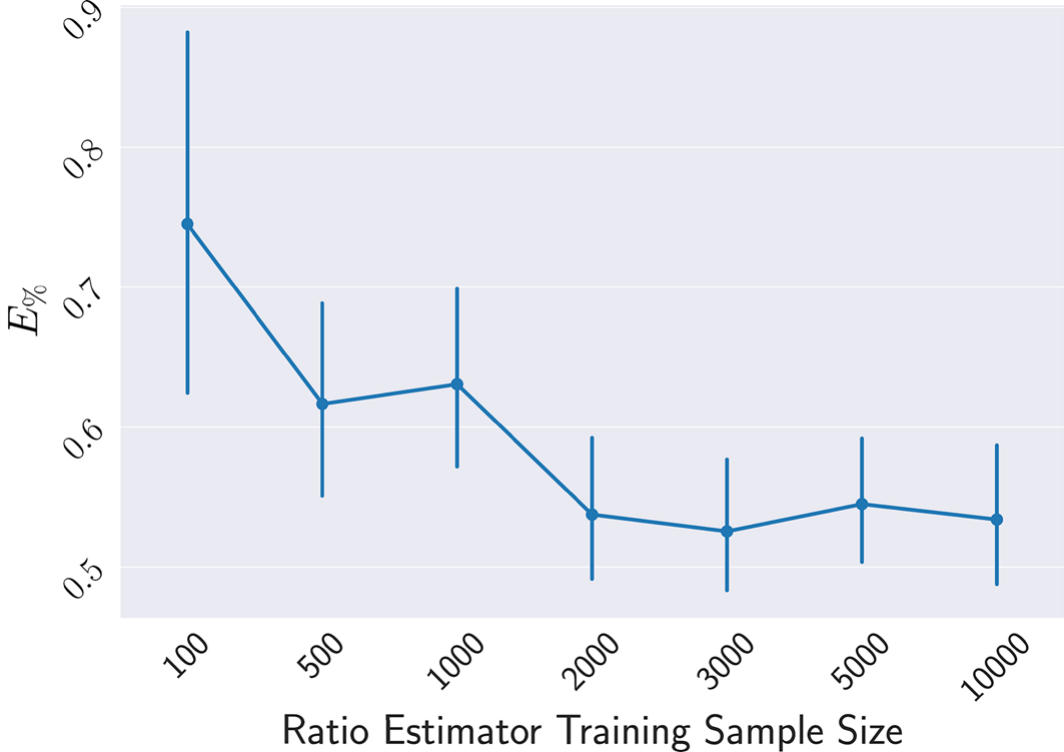
$E_\%$ error vs Number of Ratio Estimator Training Samples for Lotka-Volterra Larger *N* increases the accuracy and robustness but with diminishing returns. Selecting *M* is highly model dependent

Selecting the number of samples *M* to train the ratio-estimator is important both for the efficiency and accuracy of our method. In general, *M* depends on how sensitive the output of the model is through parameter space. If the model exhibits heavily varies throughout parameter space, *M* would naturally need to be larger to capture this. In Fig. 5, we show the performance of the approximation trained summary statistics as we change the number of initial samples *M* for the Lotka-Volterra model. While this is highly model dependent, we can see that in this case, the number of samples does not need to be high to obtain good accuracy for the summary statistic. As the approximation is relatively accurate and behavior does not rapidly change through parameter space, we only need to add full simulations from a few locations to obtain an accurate ratio estimator. After which, larger *M* only marginally changes the accuracy or robustness of the summary statistic.

In selecting $\rho$, we are trying to maximize accuracy while minimizing the number of SSA samples. An effective heuristic is to simulate a large batch of cheap, approximate trajectories, pass it through the ratio estimator, and choose $\rho$ to capture the first major mode in the distribution. For the Lotka-Volterra model, Fig. 2a would suggest to set $\rho$ around 0.01. Empirically, we find that setting the threshold quite low and effectively only correcting for the worst cases can still produce effective summary statistics. Optimal selection of $\rho$ is something to investigate in the future, as it represents a key computational trade-off. One possibility is to use Bayesian Optimization to build an estimate of $\hat{E}_{\%} = f(\rho )$ [30]. As each individual experiment may be expensive for simulation, this can lead to a more efficient hyper-parameter search technique than grid-search. Furthermore, as $\rho$ is a cut-off, computation can be accelerated for hyper-parameter tuning by saving simulations and re-using them for training summary statistics, only simulating more if necessary.

Learning an approximate ratio-estimator via binary classification, while generally an easier task than learning summary statistics, can be expensive if the parameter space is very sensitive or very high dimensional. In these cases, to distinguish between models we may need to set *M* to a large number to get the precision needed. In our examples, we are able to use a much smaller number of samples than needed to train the summary statistic. As model complexity increases, the number of training samples needed to learn a good ratio estimator will likely increase. One possibility to save some computational cost is to pre-train the first layers of the ratio-estimator to be an encoder, and then fine-tune the encoder layers to learn the summary statistic. This would act as a semi-supervised algorithm [31] that may be useful for learning a good summary statistic.

## Conclusions

We have presented a method to utilize approximate simulators together with exact simulators of discrete stochastic reaction models to train summary statistics for ABC. Using advances in Machine Learning and approximate ratio estimators, we demonstrate that when properly calibrated, we can significantly reduce the number of expensive SSA calls required for learning a summary statistic. Using four examples of reaction systems, we showed that significant computational savings can be achieved while preserving accuracy of approximate summary statistics.

In this work we have focused on utilizing only a single approximation at a time. In practice, there are numerous approximations available for the same model of varying accuracy. Extending this method to choose between different levels of approximations could further reduce the number of full SSA calls needed, even in cases where one of the approximations is sufficiently poor in all regions.

## Methods

### Approximate summary statistics

Given access to an approximate simulator $q({\tilde{X}} | \theta )$ and the full SSA simulator $p(X | \theta )$ for a given discrete stochastic biochemical system, our goal is to train a summary statistic according to (2) that utilizes as many approximate samples as possible, while mitigating the bias in doing so. We assign a computational budget of *N* total simulations and assume that the approximate simulator is much faster to simulate from than SSA. For discrete stochastic models, this assumption is accurate much more often than not. As the approximation error is often non-trivial, training a summary statistic using only approximate trajectories will likely lead to bias depending on the problem.

### Constructing an approximate dataset for training via likelihood ratios

Our approach to solving this problem is to treat each sampling step as a decision on whether the approximate simulation is sufficient. Specifically, suppose that for each sample, we draw $\theta \sim p(\theta )$ and then simulate from the approximate simulator ${\tilde{X}} \sim q({\tilde{X}} | \theta )$. The sample ${\tilde{X}}$ will induce bias in training *S*(*X*) if at $\theta$, $q({\tilde{X}} | \theta )$ is significantly different from the full SSA simulator $p({\tilde{X}} | \theta )$. Intuitively, to avoid this bias, we will need to resample, $X \sim p(X | \theta )$ and discard ${\tilde{X}}$. Computational savings will be attained if, in a substantial portion of parameter space, the approximate simulator yields a good approximation to that of SSA.

We quantify the difference between the two models using the likelihood ratio between the SSA model and the approximate model evaluated at the approximate sampled trajectory ${\tilde{X}}$ and $\theta$:

5 $$\begin{aligned} r({\tilde{X}}, \theta ) \triangleq \frac{p({\tilde{X}} | \theta )}{q({\tilde{X}} | \theta )}. \end{aligned}$$

This can be seen as conducting a hypothesis test at each step to determine whether there is sufficient evidence to distinguish which simulator the trajectory came from. If at a given $\theta$ and ${\tilde{X}}$, we cannot distinguish whether it came from the approximation or the full model, using the approximate simulation should induce little bias. If the two models produce the exact same likelihood, we would expect a value of 1, expressing indifference between the two. Most importantly, evaluating this ratio often requires simulating only from the approximate simulator, requiring a call to SSA only if we are not confident in the approximate trajectory.

Unfortunately, for discrete stochastic biochemical models, this ratio is unavailable due to the intractability of the likelihood. However, using recent advances in machine learning, we can construct powerful approximations to the likelihood ratio.
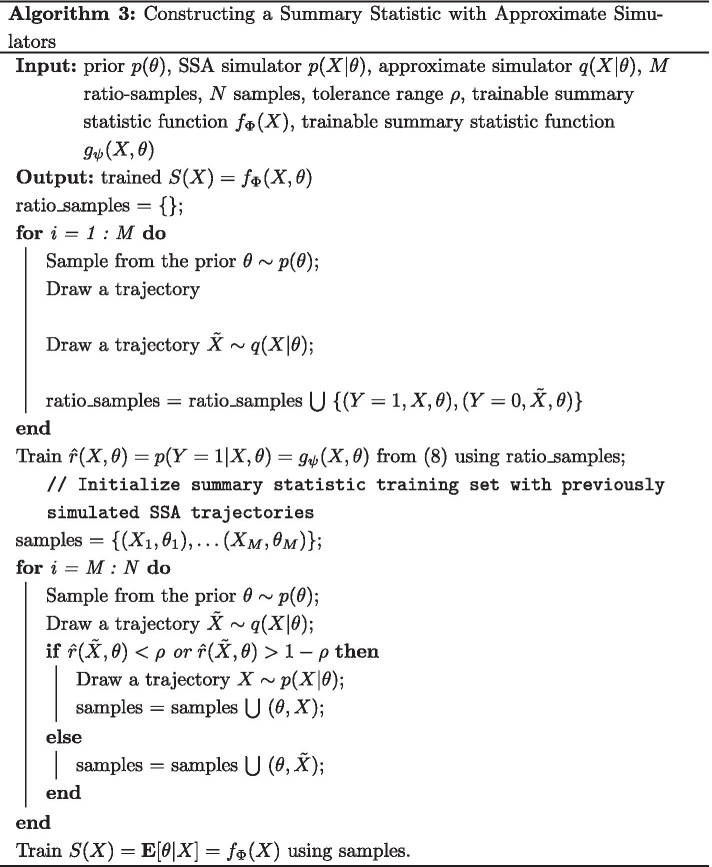


### Approximate ratio estimation

Although the likelihood ratio in (5) cannot be directly computed, recent work has shown that it can be well approximated by using a binary classifier to distinguish between samples from the two different models [22, 23, 32]. Specifically, suppose we assign labels $Y = 1$ to trajectories $X \sim p(X | \theta )$ and $Y = 0$ to trajectories from ${\tilde{X}} \sim q(X | \theta )$. If we have access to a probability $p(Y = 1 | X, \theta )$, the likelihood ratio is directly related via:

6 $$\begin{aligned}&p(Y = 1 | X, \theta ) = \frac{p(X | \theta )}{p(X | \theta ) + q(X | \theta )} \end{aligned}$$

7 $$\begin{aligned}&r(X, \theta ) \triangleq \frac{p(X | \theta )}{q(X | \theta )} = \frac{p(Y = 1 | X, \theta )}{1 - p(Y = 1 | X, \theta )}. \end{aligned}$$

As we do not have access to $p(Y = 1 | X, \theta )$, we must approximate it. Recent advances in deep learning have demonstrated how to build powerful approximations to $p(Y = 1 | X, \theta )$ despite the dimensionality of the trajectories *X*. Letting $g_{\psi }(X, \theta )$ be an arbitrarily complex function with inputs *X* and $\theta$ parameterized by $\psi$, such as a deep neural network, we can approximate the probability using:

8 $$\begin{aligned}&{\hat{p}}(Y = 1 | X, \theta ) = \phi (X, \theta ) = \frac{\exp (g_{\psi }(X, \theta ))}{1 + \exp (g_{\psi }(X, \theta )} \end{aligned}$$

9 $$\begin{aligned}&Y \sim {\text{Bernoulli}}(\phi (X, \theta )) \end{aligned}$$

with dataset

$$\begin{aligned} {\mathcal {D}} = \{(\theta _1, X_1, 1),(\theta _1, {\tilde{X}}_1, 0), \ldots , (\theta _M, X_M, 1), (\theta _M, {\tilde{X}}_M, 0)\}. \end{aligned}$$

Despite the need to train a ratio estimator, the binary classification task is easier than the regression task, allowing us to use fewer training samples than for training the summary statistic. The parameters of $g_{\psi }$ are estimated via maximum likelihood. With this initial step, we describe the full summary statistic training procedure in Algorithm 3. As $p(Y = 1 | X, \theta )$ is directly proportional to ${\hat{r}}(X, \theta )$, we use the probability as a more interpretable surrogate within the algorithm.

Implementations of this algorithm and replications of the experiments can be found at https://github.com/rmjiang7/approximate_summary_statistics.

## Supplementary Information

**Additional file 1.** Experimental Details. Details for all of the models used in the experiments along with configurations for all of the Neural Networks.

## Data Availability

Code for replicating the experiments and methods can be found at https://github.com/rmjiang7/approximate_summary_statistics.

## Abbreviations

ABC: Approximate Bayesian Computation
LFI: Likelihood-Free Inference
ODE: Ordinary differential equation
CNN: Convolutional Neural Network
SSA: Gillepsie’s Stochastic Simulation Algorithm
*E*_%_: Normalized posterior mean absolute error
$${\mathcal {U}}$$: Uniform distribution
DNN: Deep Neural Network
ABC-MCMC: Approximate Bayesian Computation-Markov Chain Monte Carlo
ABC-SMC: Approximate Bayesian Computation-Sequential Monte Carlo
