# Supplementary material for: Accelerated regression-based summary statistics for discrete stochastic systems via approximate simulators
Source: BMC Bioinformatics. 2021 Jun 23;22:339. doi: 10.1186/s12859-021-04255-9 (PMC8220802; doi:10.1186/s12859-021-04255-9)
Supplement: Supplementary file 1 — Additional file 1. Experimental Details. Details for all of the models used in the experiments along with configurations for all of the Neural Networks. [file 12859_2021_4255_MOESM1_ESM.pdf]

Additional File 1: Experimental Details

Richard Jiang Fredrik Wrede Prashant Singh Andreas Hellander
Linda Petzold

1 Pure-Birth Process

The Pure-Birth Process is represented as

$$\phi \xrightarrow{k} S.$$

As this is simply a homogenous Poisson process, we can evaluate the likelihood of an observation at any time t as

$$P(S(t)|S(0), k) = \frac{k^{S(t)-S(0)}e^{-k}}{(S(t)-S(0))!}.$$

We assign prior $k \sim \mathcal{U}(0, 10000)$ and observations are made of S at times $t = \{1 : 100 : 1\}$. We train the ratio estimator using $M = 300$ samples from both the SSA and the Tau-Leaping approximation. The summary statistic is trained using $N = 5000$ samples. The posterior in the main text is obtained using $k = 2432$.

2 Lotka-Volterra Stochastic Oscillator

The Lotka-Volterra Stochastic Oscillator is described by

$$S_1 + S_2 \xrightarrow{k_1} 2S_1 \qquad S_1 \xrightarrow{k_2} \phi$$
$$S_2 \xrightarrow{k_3} 2S_2 \qquad S_1 + S_2 \xrightarrow{k_2} S_2.$$

We assign the following priors

$$\log(k_1) \sim \mathcal{U}(-6, 2) \qquad \log(k_2) \sim \mathcal{U}(-6, 2)$$
$$\log(k_3) \sim \mathcal{U}(-6, 2) \qquad \log(k_4) \sim \mathcal{U}(-6, 2),$$

and observations are made of both S_1 and S_2 at times $t = 0 : 30 : 0.2$, for a total of 150 time steps. We train the ratio estimator using $M = 3000$ samples from both the SSA and the ODE approximation. The summary statistic is trained using $N = 100000$ samples. The posterior in the main text is obtained from $\mathbf{k} = [0.01, 0.5, 1.0, 0.01]$, giving oscillatory behavior.

3 Genetic Toggle-Switch

The Genetic Toggle-Switch is described as

$$\phi \xrightarrow{\frac{\alpha_1}{1+V^{\beta}}} U \qquad \phi \xrightarrow{\frac{\alpha_2}{1+V^{\gamma}}} V$$
$$U \xrightarrow{\mu} \phi \qquad V \xrightarrow{\mu} \phi.$$

We assign the following priors

$$\alpha_1 \sim \mathcal{U}(0,3) \qquad \alpha_2 \sim \mathcal{U}(0,3)$$
$$\beta \sim \mathcal{U}(0,3) \qquad \gamma \sim \mathcal{U}(0,3) \qquad \mu \sim \mathcal{U}(0,3),$$

and observations are made of both U and V at times $t = 0 : 50 : 0.25$, for a total of 200 time steps. We train the ratio estimator using $M = 5000$ samples from both the SSA and the Tau-Leaping approximation. The summary statistic is trained using $N = 100000$ samples.

4 Vilar-Oscillator

The Vilar-Oscillator is described as in (1). We assign the following priors to the parameters:

$$\alpha_A \sim \mathcal{U}(0,80), \quad \alpha'_A \sim \mathcal{U}(100,600), \quad \alpha_R \sim \mathcal{U}(0,4), \quad \alpha'_R \sim \mathcal{U}(20,60),$$
$$\beta_A \sim \mathcal{U}(10,60), \quad \beta_R \sim \mathcal{U}(1,7), \quad \delta_{MA} \sim \mathcal{U}(1,12), \quad \delta_{MR} \sim \mathcal{U}(0,2),$$
$$\delta_A \sim \mathcal{U}(0,3), \quad \delta_R \sim \mathcal{U}(0,0.7), \quad \gamma'_A \sim \mathcal{U}(0.5,2.5), \quad \gamma_R \sim \mathcal{U}(0,4),$$
$$\gamma_C \sim \mathcal{U}(0,3), \quad \theta_A \sim \mathcal{U}(0,70), \quad \theta_R \sim \mathcal{U}(0,300),$$

and observations are made of species C, A, and R at times $t = \{0 : 100 : 1\}$. Most parameters are poorly identified under these settings (2). To simulate more realistic conditions, we also perturb the ODE trajectories with log-normal noise. This prevents the ratio estimator from overfitting to the smooth ODE solutions, as mentioned in the main text.

We train the ratio estimator using $M = 10000$ samples from both the SSA and the noise added ODE approximation. The summary statistic is trained using $N = 200000$ samples to more thoroughly explore the high dimensional parameter space.

5 Neural Network Architectures

Table 1: Neural Network Architectures

	Ratio Estimator	Summary Statistics
Pure-Birth	CNN	CNN
Lotka-Volterra	MLP(50,50)	CNN
Genetic Toggle Switch	CNN	CNN
Vilar Oscillator	CNN	CNN

In Table 1 we list the details to train the approximate ratio estimator and the summary statistic. The referenced CNN follows the construction from (2) while the referenced Multi-Layer Perceptron (MLP) specifies the number of hidden neurons with ReLU activation functions. For all experiments, we implement the model in PyTorch and use the Adam Optimizer with an exponential learning rate scheduler to train the Neural Networks.

References

[1] Vilar, J.M., Kueh, H.Y., Barkai, N., Leibler, S.: Mechanisms of noise-resistance in genetic oscillators. Proceedings of the National Academy of Sciences **99**(9), 5988–5992 (2002)

[2] Åkesson, M., Singh, P., Wrede, F., Hellander, A.: Convolutional neural networks as summary statistics for approximate bayesian computation. arXiv preprint arXiv:2001.11760 (2020)
